# Navigating technology-facilitated abuse: A participatory action research study with young adults living with HIV and key populations in Kenya

**DOI:** 10.1371/journal.pgph.0007060

**Published:** 2026-08-14

**Authors:** John N. Macharia, James Kiilu, Simon Odiwuor, Winnie Gift, Timothy Wafula, Bernard Koomson, Sara L.M. Davis

**Affiliations:** 1 Research, Monitoring and Evaluation Department, Kenya Legal & Ethical Issues Network on HIV and AIDS (KELIN), Nairobi, Kenya; 2 Outreach Department, Integrated Cancer Research Foundation, Nairobi, Kenya; 3 HIV, TB and Key Population Thematic Area, Kenya Legal & Ethical Issues Network on HIV and AIDS (KELIN), Nairobi, Kenya; 4 Centre for Interdisciplinary Methodologies (CIM), University of Warwick, Coventry, United Kingdom; PLOS: Public Library of Science, UNITED STATES OF AMERICA

## Abstract

For young adults living with HIV and key populations, digital platforms are essential tools for accessing health information, services, and navigating stigmatized health needs. However, these technologies expose them to digital harms such as technology-facilitated abuse (TFA). Research on TFA has largely focussed on gender-based violence with limited exploration of experiences of criminalised and stigmatised populations. This paper reports findings from Kenya as part of the Digital Health and Rights Project (DHRP), a four-country study conducted in Colombia, Ghana, Kenya, and Vietnam. The study examined how young adults living with HIV and key populations experience TFA. Data were collected in Nairobi, Mombasa, Kitui, and Migori counties through 16 focus group discussions with 109 young adults aged 18–30 years, including people living with HIV, female sex workers, gay men and other men who have sex with men, and transgender participants, alongside eight in‑depth interviews and six key informant interviews with policymakers, health professionals, and civil society leaders. Data were thematically analysed. TFA included cyberbullying, hacking, blackmail, impersonation, malicious reporting of accounts, and non‑consensual sharing of images or private information. Perpetrators included family members, partners, friends, and strangers, often linked to participants’ HIV status, sexuality, or online visibility. TFA caused significant emotional, social, and behavioural harms. Participants emphasized the need for digital literacy, stronger data protection, and a responsive criminal justice system. Based on these findings, we propose an expanded intersectional definition of TFA that extends beyond gendered framing to explicitly recognise HIV status, sexual orientation, and gender identity and expression as axes of vulnerability. Our findings highlight the urgent need for rights-based digital governance that moves the burden of protection from individuals to systems, including through decriminalisation, to protect digital rights and wellbeing of young adults living with HIV and key populations in Kenya.

## Introduction

People living with HIV and key populations key populations (that is, gay men and other men who have sex with men, sex workers, transgender people, people who inject drugs and incarcerated individuals as defined by WHO consolidated guidance) continue to experience overlapping forms of social stigma, exclusion, and marginalization - and often lack adequate access to health and social services due to such stigma and criminalisation [1,2]. For many, digital platforms have become essential tools for accessing and sharing health information, building peer support, and connecting with the community [3]. As Davis et al. note, these platforms provide discreet ways to navigate stigmatized health needs, such as sexual and reproductive health, which are often inaccessible offline; however, they can also expose users to significant risks [4].

Amid these growing digital harms, young adults living with HIV (young adults living with HIV) and key populations have called for a greater role in digital governance to ensure their rights are upheld [4]. Community organizations representing key populations report that digital health infrastructures are often being designed and implemented with minimal consultation, despite the high stakes for privacy [5]. Although global HIV and human rights frameworks call for the meaningful involvement of people living with HIV and key populations in policy and governance, evidence consistently documents their underrepresentation in digital health and governance [6,7]. These governance gaps, coupled with emerging forms of online harm, have highlighted the growing phenomenon of Technology‑Facilitated Abuse (TFA).

The conceptualization and scope of TFA remain contested, with a range of definitions and terminologies used across jurisdictions, practices, and fields of knowledge. To address this fragmentation, UN Women convened a group of experts who reached a consensus definition of TFA as “any act that is committed, assisted, aggravated, or amplified by the use of Information and Communication Technologies (ICTs) or other digital tools, and that results in or is likely to result in physical, sexual, psychological, social, political, or economic harm, or other infringements of rights and freedoms.” [8]

The complexity in conceptualizing TFA is further illustrated by a global Delphi study involving experts from diverse fields, which revealed significant variations in how TFA is understood and labelled [9]. The study reached a consensus that TFA involves the misuse of digital tools to cause harm, emphasizing the behaviour of perpetrators, the impact on victims, the role of consent, and the broader social context. However, there was no consensus on whether TFA should be defined narrowly, such as limited to gender-based or intimate partner abuse, or more broadly to include strangers, institutions, and systemic digital harms. For this study, we adopt the definition of TFA articulated by UN Women.

TFA has been documented across a wide range of contexts, including intimate partner relationships, and sexual- and gender-based violence [9,10]. In these settings, TFA is often driven by power imbalances, structural conditions, and broader ecosystems of sexism and patriarchy [11]. It is frequently motivated by coercive control, with perpetrators intending to intimidate and assert dominance over their victims [12,13].

Despite the recognition of TFA as a global health and human rights concern, most existing research has focused on TFA in the context of intimate partner violence, gender-based violence, and sexual exploitation, with young women as the primary focus of analysis [14–17]. Even broader handbooks on technology‑facilitated violence and abuse tend to frame TFA as a spectrum of gender‑based and sexual violence, situating it within established feminist and gender-based violence (GBV) frameworks [18].

Within this dominant framing, several gaps are evident. First, there has been a limited exploration of TFA among men, Lesbian, Gay, Bisexual, Transgender, Intersex, Queer/Questioning, and others (LGBTIQ+) populations, people living with HIV, people with disabilities, and other minority groups [19,20]. TFA among young adults living with HIV and key populations in criminalized and marginalized contexts remains underexamined, despite repeated calls to expand TFA research to structurally vulnerable and minority populations in low- and middle-income countries (LMICs) [20]. Although sexual and gender minority groups experience higher rates and distinctive patterns of TFA, they remain underrepresented in the broader literature [19]. Secondly, TFA research is heavily concentrated in high income Western countries, with limited representation from LMICs, particularly in sub-Saharan Africa [21–23]. This concentration constrains understanding of TFA as experienced in settings shaped by legal criminalization, structural and policy neglect, and persistent digital divides, limiting the development of contextually appropriate responses in LMICs.

In Kenya, young adults living with HIV and key populations continue to face persistent stigma, surveillance, and legal uncertainty [24,25]. Emerging studies have examined how these structural conditions shape engagement with digital health and online platforms; however, the evidence base remains fragmented and limited [4,26]. The criminalization of same-sex relations under Kenya’s Penal Code, for example, makes being outed online a serious threat to the safety of gay men and other men who have sex with men [27]. For these communities and other key populations, digital platforms can become tools of control, coercion, and harm [28]. As a result, many individuals navigate a delicate balance between visibility and survival, often leading to self-censorship or withdrawal from essential online health spaces [29]. As digital technologies become increasingly central to HIV programming, particularly HIV prevention interventions, there is a critical need to explore how TFA manifests within these populations [30]. This study, part of a broader transnational participatory action research (T-PAR) initiative conducted in Colombia, Ghana, Kenya and Vietnam, documents experiences of TFA among young adults living with HIV, female sex workers, gay men and other men who have sex with men and transgender people across four counties in Kenya.

The study is grounded in a rights-based approach that prioritizes the voices and lived experiences of young adults living with HIV and key populations, who are often excluded from policy discussions on digital governance. These populations were actively involved in the study through the leading role of national investigators at a Kenyan civil society organisation, and through the formation of a Kenya Community Advisory Team (K-CAT), which sought to ensure meaningful participation of study populations throughout the research process. By centering lived realities, the study illuminates the complex interplay between digital transformation, structural inequalities, and human rights risks in LMICs. The findings contribute to the growing evidence base needed to inform equitable digital health practices, strengthen privacy protections, and shape inclusive digital governance frameworks.

## Methods

### Ethics statement

Ethical approval was obtained from the Humanities and Social Sciences Research Ethics Committee, University of Warwick [HSSREC 15/23–24] and the AMREF Ethics and Scientific Review Committee, Kenya [ESRC P1582/2023]. The study also received a national research license in Kenya from the National Commission for Science, Technology and Innovation [NACOSTI/P/24/34618]. Participation was voluntary, with written informed consent obtained from all participants prior to data collection, witnessed by a non-research-team member. The research team took special care to minimize risks for participants, who face heightened vulnerability to stigma, discrimination, and potential legal or social harm, by conducting interviews in safe and private environments selected in consultation with the sampling organizations [2]. To safeguard confidentiality, participants were invited to choose pseudonyms; only researchers conducting the interviews were present; and all transcripts were fully anonymized, encrypted, and stored on password-protected servers accessible only to authorized researchers. Data collection was guided by trauma-informed approaches, and consistent with best practices for qualitative research with potentially vulnerable populations, referral pathways for psychosocial support were established to address distress or the reactivation of traumatic experiences [31].

### Study design and setting

The study applied a qualitative, Transnational Participatory Action Research (T-PAR) approach to explore young adults’ experiences of TFA [32]. PAR enables the meaningful involvement of affected communities as active partners in the research process. Our transnational approach emphasized cross‑national collaboration, shared decision‑making, cross‑learning, and advocacy for global impact across Colombia, Ghana, Kenya and Vietnam.

As part of this commitment, Community Advisory Teams (CATs) were established in each country, consisting of 12–14 members from diverse backgrounds, including human rights activists, representatives of key populations, and civil society leaders. In Kenya, the CAT comprised of 12 members and was chaired by a national health and human rights organization, KELIN, which led the research in Kenya and collaborated with researchers from the other countries in project management, research, advocacy, and governance. The CAT guided the research process by identifying priority focus areas, advising on methods, reviewing research tools, and leading participant recruitment. Some CAT members also served as research assistants conducting Focus Group Discussions (FGDs), In-depth interviews (IDIs) and Key Informant Interviews (KIIs). The CAT also elected representatives to the project’s transnational Steering Committee to oversee the four-country project. This study is co-authored with three members of the Kenya CAT (JK, WG, SO).

This approach enables the co-production of knowledge, shared decision-making, and an emphasis on action-oriented outcomes, making it well suited for exploring sensitive issues related to health and rights in marginalized communities [33,34]. Evidence suggests that youth-led participatory action research enhances agency, ethical responsiveness, and contextual relevance in sexual and reproductive health and rights research, while T-PAR in digital health and rights can create “brave spaces” for participation and challenge inequitable data practices [28,35,36].

### Study sites

The study was conducted in four Kenyan counties: Nairobi, Mombasa, Kitui, and Migori, selected to represent diverse geographic and socioeconomic contexts, including urban (Nairobi and Mombasa), peri-urban (Migori), and rural (Kitui) settings. While internet penetration in Kenya has improved overall, rural areas continue to experience persistent gaps in connectivity, infrastructure, and digital literacy compared to urban centres [37]. According to the Communications Authority of Kenya, internet usage stands at 66.7% in Nairobi and 46.9% in Mombasa, compared with 30.4% in Migori and 26.2% in Kitui [38]. These counties were selected to capture such variations in digital access and experience. Selection was also informed by the presence of organized key populations groups which facilitated participant recruitment. Collectively, the counties represent distinct regions of Kenya with substantial youth populations and varying stages of digitization, enabling exploration of how TFA manifests across urban–rural contexts.

### Study population, sampling and recruitment

The study population included young adults aged 18–30 years, specifically individuals living with HIV, gay men and other men who have sex with men, female sex workers and transgender people. The age range was defined by the research consortium and aligns with definitions commonly used in global HIV-led youth networks and advocacy movements, which often categorize “young adults” as individuals 18–30 years [39,40]. Participants were eligible if they: (i) were aged 18–30 years; (ii) self-identified as a member of one of the four target population groups; and (iii) resided in one of the four study counties.

A total of 109 young adults were recruited between 20^th^ May 2024 and 20^th^ July 2024. Study participants were recruited by K-CAT members, trained research assistants, community-based organizations (CBOs), and local peer educators working in close consultation with the KELIN, a national health and human rights group implementing the project in Kenya. Purposive and snowballing sampling strategies were employed, with recruitment primarily facilitated through Key population organizations that have established trust and networks within young adult communities. Purposive sampling guided the initial identification of participants to ensure representation across the four target population groups and study counties, while snowball sampling was subsequently used to expand the sample within each population group, with initial participants referring peers who met the eligibility criteria and were willing to participate. Stigma associated with key populations often makes individuals reluctant to disclose sexual identity or behavior, limiting the feasibility of constructing of a comprehensive sampling frame [41]. The combined use of purposive and snowballing sampling strategies is thus effective in recruiting such populations by leveraging trusted channels, such as community organizations [41,42].

The study conducted a total of 16 FGDs (four per county) between May and June 2024. The FGDs were stratified by population group, with separate sessions for young adult women living with HIV, young adult men living with HIV, female sex workers, and gay men and other men who have sex with men in each county. Each FGD included 6–9 participants, yielding a total sample of 109 young adults across all study sites. In addition to the FGDs, eight IDIs were conducted to further explore key themes that emerged during the group discussions. Participants for the IDIs were purposively selected from among FGD participants, based on the research team’s assessment of their ability to provide deeper insights into the issues discussed. In addition, six KIIs were conducted with policymakers, health professionals, and civil society leaders, selected for their expertise in digital rights, HIV, and key population issues in Kenya, to provide institutional and governance perspectives on TFA.

### Research team

Interviews and FGDs were conducted by researchers with graduate-level training in social sciences, public health, law and human rights, employed at academic institutions, NGOs, and community-based organisations. The team included male and female facilitators, all of whom had prior training in qualitative methods, participatory action research, and trauma-informed approaches.

### Data collection

Interviews were conducted in English, Swahili, Sheng, Kamba, and Dholuo by researchers fluent in these languages using semi-structured interview guides that had been piloted. FGDs lasted approximately 90–120 minutes while IDIs and KIIs lasted between 30 and 45 minutes. All interviews were transcribed verbatim, translated into English, and cross-checked for accuracy. Field notes were taken during and immediately after each data collection session. Data were then analyzed thematically using Dedoose software. No repeat interviews were conducted.

### Data analysis

Data analysis was carried out collaboratively by researchers from each national team together with the principal investigator, following the Collaborative Qualitative Analysis approach developed by Richards and Hempill [43]. The process combined open and axial coding to identify key themes and patterns within the dataset, using Dedoose, a cloud-based qualitative analysis platform. Researchers drafted analytic memos identifying generative themes, which were then discussed in weekly consortium meetings. After coding a representative portion of data, the team developed a preliminary codebook and tested it against uncoded transcripts. Following collaborative revisions, the final codebook was applied to the entire dataset. Each transcript was coded by one researcher and independently reviewed by a second; any discrepancies were documented in a shared online journal, discussed during team meetings, and resolved by a third researcher to ensure consistency and reliability.

National research teams presented draft findings and recommendations to CATs and study participants during online validation meetings, incorporating feedback into the final analysis. The consolidated findings and recommendations were subsequently presented to and approved by the full consortium, including CAT representatives, during a workshop in Cape Town, South Africa (January 2025) for inclusion in national and global advocacy plans.

### Inclusivity in global research

Additional information regarding the ethical, cultural, and scientific considerations specific to inclusivity in global research is included in the Supporting Information (S1 Checklist). This study is reported in accordance with the Consolidated Criteria for Reporting Qualitative Research (COREQ); the completed checklist is provided in S2 Checklist.

## Results

### Participant distribution

A total of 109 individuals aged 18–30 years participated in the study across the four counties. Table 1 presents the distribution of the participants by county and self-reported gender.

**Table 1:** Participant Distribution by county and self-reported gender.

County	*Male*	*Female*	*Trans*	*Non-binary*	*Total*
Kitui	13	14	0	0	**27**
Migori	14	13	0	1	**28**
Mombasa	12	4	5	3	**24**
Nairobi	14	16	0	0	**30**
**Total**	**53**	**47**	**5**	**4**	**109**

TFA emerged as a widespread and deeply gendered form of digital harm, amplifying participants’ existing offline vulnerabilities. Among the 109 participants, 53 reported experiences of TFA, with the highest prevalence among female, non-binary, and transgender participants. Thematic analysis identified multiple, overlapping forms of TFA, their mental and physical impacts, and participants’ strategies for resistance and coping. These abuses were enabled and intensified by systemic gaps in data protection, platform governance, and digital literacy.

### Forms of TFA

Participants reported a wide range of TFA, from interpersonal attacks such as cyberbullying to more technical harms such as hacking, impersonation, and fraud. These forms of TFA often overlapped and were shaped by participants’ identities, HIV status and level of digital literacy, frequently intersecting with broader practices of digital surveillance.

#### Cyberbullying.

Cyberbullying was the most reported form of TFA. Participants described being targeted on Facebook, X (Twitter), TikTok, and Instagram when sharing content related to sexual and reproductive health, HIV, or LGBTQ+ identities. Reported harms included verbal abuse, sexual harassment, circulation of intimate or manipulated images, and threats of physical violence. Cyberbullying was often experienced as a form of social control, aimed at restricting expression and the sharing of certain health-related topics. As a 24-year-old non-binary participant from Mombasa (2024-MSA-FGD14–92) explained:

*Someone tells you don’t talk about this topic. Say you’re talking about PrEP, STIs, condoms. So, you can’t educate other people just because of cyberbullying and the foul words which they are using…you don’t have that courage to share, even if you’re saying something right, they will use that against you*

Some participants described experiencing cyberbullying not because of what they posted but solely on the basis of their HIV status. As a 24-year-old female living with HIV from Mombasa (2024-MSA-FGD16–104), recounted:

*I am a victim. There is someone, I don’t know where they got the information that I am HIV positive. So, they sent me a text and what was in the text was so hurtful I cannot talk about them here.*

Cyberbullying also included the non‑consensual use of social media photos to generate memes and public ridicule, and in some cases escalated to death threats and account closure, prompting participants to withdraw from platforms. A 24-year-old non-binary participant from Mombasa (2024-MSA-FGD14–92) described:

*I shared a cut scene [a short segment of a personal experience]…So, people started talking, “you have a few days to live” to the point that I closed my account*

#### Hacking and fraud.

Participants described hacking of devices and social media accounts, often followed by financial fraud or the unauthorized posting of explicit content. A 23-year-old female participant from Nairobi (2024-NBO-FGD1–5) recounted how a hack led to reputational damage:

*They hack your account, streaming, they start posting, pornography those things... people who follow you start to judge you, wondering whether you changed or you just became a pornographer*

Similarly, a 22-year-old participant from Kitui (2024-KTU-FGD12–85) described her experience as a victim of hacking and fraud:

*My account was hacked by somebody who was passing false information saying that my parents had an accident and died... people sent a lot of money to that number*

#### Sharing of images/ involuntary disclosures.

Participants reported non‑consensual sharing of photos and screenshots, often intended to disclose sexual identities or HIV status without their consent. These incidences were frequently facilitated by interpersonal surveillance and the use of shared devices. A 28-year-old female participant from Kitui (2024-KTU-FGD9–64) described her experience:

*I had not disclosed my status, nobody knows if I’m HIV positive. My friend gets my phone and accesses our groups, they get that information. Some take screenshots, people start isolating you…at long last you come to know that they already know your status*

#### Blackmail and extortion.

Participants reported that blackmail and extortion commonly involved threats to disclose sexual orientation or HIV status unless they complied with demands, often for money. A 23-year-old male participant from Migori (2024-MIG-FGD7–49) described such cases:

*Your partners has your information about your sexual orientation. When you are not in good terms, he can just share the information in public where it is impossible for you to catch up with the thing. It becomes difficult living while knowing the information is out there.*

Others reported being recorded during online interactions and then threatened with the public release of videos unless they sent money or additional content.

#### Malicious reporting of accounts.

Participants described coordinated reporting of their accounts, particularly when posting health‑related or queer content, as a tactic to silence and punish them. Such reports could lead to temporary suspensions, content removal, or fear of permanent bans. A 24-year-old participant from Mombasa (2024-MSA-FGD13–86) described her experience:

*See if a person doesn’t like you on TikTok, it’s very easy. She will just decide to report your account and tell her friends to do the same. Literally, you’ve done nothing. I feel they should have more measures, a clear process. I feel people are abusing the fact that I can report.*

#### Impersonation.

Impersonation emerged as a targeted and particularly harmful form of TFA, especially affecting gay men and other men who have sex with men. Perpetrators used stolen photos and names to create fake profiles, often on dating apps, to solicit money, entrap, or blackmail others. A 26-year-old non-binary participant from Mombasa (2024-MSA-FGD15–99) described this practice:

*Queer individuals’ experience phishing (a type of cyberattack where scammers trick people into revealing sensitive information) on a day to day especially on queer apps (such as Grindr). Stealing your identity, they take some of your pictures from social media and they open a dating app and they use that information to get people, to get money, to blackmail.*

Shared phones and device use were reported to increase this risk, as acquaintances accessed existing accounts and then posed as the account holder to contact “customers” or sexual partners.

### Perpetrators of TFA

Participants described TFA as perpetrated by a range of actors, including family members, intimate partners, peers, and friends with access to personal devices or online groups, and strangers. While abuse occurred across all these relationships, most reported incidents were perpetrated by strangers. Stranger‑perpetrated TFA included cyberbullying by anonymous users, impersonation, phishing, account hacking, blackmail by people met online, and entrapment by individuals posing as clients or community members. Participants described receiving abusive messages and attacks in comment sections from people they had never met. The anonymity afforded by digital spaces often left victims with little recourse. A 23-year-old female participant from Nairobi (2024-NBO-FGD1–5) explained:

*I don’t know who they are, but they hack your account, start posting…you don’t know the person who hacked you, you don’t know the actions you should take against them.*

Participants also reported that some perpetrators acted as collectives, coordinating harassment through public platforms. A 29-year-old female from Mombasa (2024-MSA-FGD13–91) described:

*There’s something called KOT (Kenyans on Twitter). Those people are ruthless. Those people can shred you like a shredded chicken.*

While stranger-perpetrated abuse was most common, TFA by family members, intimate partners, and friends was reported as particularly distressing due to prior trust and proximity. Family-perpetrated TFA often involved the exposure or threatened exposure of sensitive content. As a 23-year-old male participant from Kitui (2024-KTU-FGD10–69) explained:

*Last week I had a case, where one peer came across one of the family members in the MSM group. And the guy was threatening the other one saying, ‘Now try me and I will post the nudes you sent to that group.*

Intimate partners and ex-partners engaged in online stalking, blackmail, and image-based abuse, using digital tools to maintain control or humiliate participants after relationships ended. A 24-year-old female participant from Migori (2024-MIG-FGD5–31) said:

*I had an ex, and I still had the number. So I go on posting on Facebook as usual, memes and whatever. The next thing, someone posts on status… the way he puts it, it indicates that I am the target. From that I could tell that he was busy stalking me.*

Friends and peers sometimes exploited personal trust or shared group membership for blackmail. A 24-year-old male participant from Migori (2024-MIG-FGD7–47) reported:

*You see on this blackmail, there is a time a friend of mine knew my secrets, so he wanted me to do him one favor so as not to expose my secrets.*

### Impact of TFA on young adults living with HIV and Key Populations

Across the four counties, participants described TFA as having serious emotional, social, and physical consequences. Emotional impacts included lowered self‑esteem, fear, anxiety, isolation, stigma, depression, and suicidal ideation. Behaviourally, TFA led to self‑censorship, withdrawal from digital communities, deletion of accounts, and avoidance of online health discussions. Socially and materially, participants reported damaged relationships, disownment by parents, physical assault, and economic losses. There were instances of online threats escalating into physical violence. A 27-year-old trans participant from Mombasa (2024-MSA-FGD14–96) described the threat of violence following a post on TikTok:

*A Boda guy (motorcycle taxi driver) had taken me from work to my house, after he dropped me, I think he went through his TikTok. He saw me on the FYP- for you page. He started attacking me, saying, “You are the one I just carried on my motorcycle, you are a homosexual!” I panicked*

Participants linked TFA to emotional distress and, in some cases, suicidal thoughts as the 21-year-old female participant from Nairobi (2024-NBO-FGD1–7) describes:

*When the information is leaked, it will lead to being abused verbally…that is going to hurt you mostly emotionally. And that is going to lead to suicidal thoughts and all that.*

TFA also reshaped participants’ digital engagement, particularly around HIV and sexual health. Many avoided commenting on platforms or sharing health information, while some deleted accounts entirely. A 24-year-old non-binary participant from Mombasa (2024-MSA-FGD14–92) explained:

*You find most of the people don’t share that information through the internet because you’ll find posting this information will expose you to cyberbullying. Most of us have this information but we don’t share it.*

For gay men and other men who have sex with men, this fear extended even to private health‑related searches. A 27-year-old male participant from Nairobi (2024-NBO-FGD3–16) said:

*People would search on Google and get results but now they are not...there was anti-LGBTQ so with that most of the community members are scared of searching health-related issues because they might search anal warts and the government is watching*

### Everyday coping and acts of resistance to TFA

Participants described a range of strategies to manage or resist TFA. Common responses included blocking abusive users, reporting accounts, deleting profiles, tightening privacy settings, and self‑censoring content. Some participants chose direct confrontation or education before disengaging. A 28-year-old female participant from Mombasa (2024-MSA-FGD13–90) explained:

*You come to me whatever it is that I’ve posted, you want to bully me? I’m also a bully. You’ll bully me. I’ll bully you.*

Others sought to educate perpetrators, particularly when abuse came from religious or conservative contacts reacting to sex work or LGBTQ+ content. A 28-year-old female participant from Mombasa (2024-MSA-FGD13–89) described:

*Before I block you, I’ll try to inform you. I’m a sex worker, also part of the LGBTQ (population). It’s hard when I post something, and I have religious people viewing my status. You can imagine the backlash that I used to get. So rather than block you,... we will sit somewhere we can discuss. If I notice that you are someone who doesn’t get anything [then] I will block you.*

A 26-year-old non-binary participant from Mombasa (2024-MSA-FGD15–99) reported using platform features to limit certain users:

*My experience since nowadays social media apps have more restrictive features, [is that] you can restrict certain words so people cannot comment offensive words. There are many ways you can counter the cyber bullying these days.*

Very few participants engaged the criminal justice system. Many expressed deep mistrust of the police and feared further harm, given the criminalized nature of their identities and work. Among those who did report, support was minimal. Instead, some participants turned to community‑based mechanisms, including seeking help from paralegals.

### Facilitators of TFA: Low digital literacy and poor data protection

Participants identified limited digital literacy and weak data protection as key facilitators of TFA. Rather than isolated breaches, they described a systemic environment where personal information could be easily accessed, shared, and misused without consent or accountability. Concerns extended to third‑party data sharing, misinformation, and emerging AI‑driven risks. Several participants emphasized the need for government‑led digital literacy to help individuals secure their accounts and respond quickly to potential breaches. A 29-year-old female participant from Kitui (2024-KTU-FGD9–59) said:

*Government should teach us on issues surrounding IT. If someone wants to access your information, you can change the password fast before they get your information. We should be more informed about IT.*

Another 23-year-old male participant from Kitui (2024-KTU-FGD10–70) stressed the importance of digital literacy and awareness:

*The public should be educated on the matter of privacy...You have the right to sue the person who leaks your information.*

Participants also noted that Kenya’s regulatory environment was weak compared to regions with stricter enforcement, leaving individuals and public systems vulnerable to data breaches. They stressed that digital health initiatives must integrate digital security and community awareness. A 29-year-old male participant from Nairobi (2024-NBO-FGD3–23) explained:

*You cannot speak about digital healthcare without putting [in] digital security…If you are going to train the entire community on something, you have to include digital security together. How do they know this is the right information?*

## Discussion

As the internet penetration in Kenya continues to grow rapidly, increasing numbers of young people are turning to digital platforms to access health information and peer support [4,44]. A previous T-PAR project conducted in Ghana, Kenya and Vietnam by the Digital Health and Rights Project Consortium, found that young people reported using digital platforms to access sexual and reproductive health and HIV-related information, often due to stigma or barriers in formal healthcare settings [4]. The study highlighted both the benefits and risks of digital transformation of health, including empowerment, misinformation, online abuse, and surveillance. Building on this earlier work, the present paper offers a more comprehensive exploration of digital harms experienced by young adults in Kenya.

### Intersecting vulnerabilities in the digital age

In this study, we documented multiple forms of TFA experienced by young adults living with HIV and key populations. These experiences contribute to the growing global evidence that, while digital platforms offer important opportunities for health communication and service delivery, they also introduce new forms of risk and harm [4]. As digital tools become increasingly integrated into HIV programming, it is essential to understand how TFA manifests within these communities [30]. Such understanding is critical in ensuring that digital health platforms are secure, equitable, and inclusive for those who rely on them most.

We documented that TFA against young adults living with HIV and key populations was primarily shaped by two intersecting factors and occurred across two layers of vulnerability. The intersecting factors that shaped the experience of TFA were sexual/gender identity and HIV status. Participants reported being attacked online primarily because they identified as LGBTQ+ or were living with HIV. Being a young adult living with HIV or a member of key populations constituted the first layer of vulnerability. We also documented the gendered nature of TFA, which formed the second layer of vulnerability. TFA was most commonly reported by, and occurred among, female, non-binary, and transgender participants. These findings are consistent with a study by Turner et al., which found that TFA was more common among sexual and gender minority youth, who had 2–8 times greater odds of exposure compared to heterosexual men [45]. In addition, females had a higher risk of exposure regardless of sexual identity. Our study presents a double layer of vulnerability to TFA among young adults living with HIV and key populations reflecting an extension of the offline vulnerabilities they already face.

As stated earlier, this study draws on the UN Women definition of technology‑facilitated violence against women and girls [8]. While this definition provides an important foundation for understanding the gendered nature of digital harms, our findings demonstrate that the scope of those affected extends beyond cisgender women and girls. In light of our findings, we propose an expanded, intersectional framing of TFA that explicitly recognizes sexual orientation, gender identity and expression, and HIV status as axes of vulnerability. Broadening the analytical frame in this way allows for a more accurate understanding of how digital harms operate across intersecting systems of gender inequality, heteronormativity, and HIV‑related discrimination.

While some perpetrators of TFA were known to participants, most were reported to be strangers. The Delphi Global Study of TFA noted that although TFA can involve personal and professional relationships, it may also be perpetrated by strangers [9]. Our study found that stranger-perpetrated TFA was the most commonly reported pattern among young adults living with HIV and key populations. Although interpersonal TFA remains a significant concern within personal relationships, it necessitates distinct and actionable recommendations for effective intervention [46]. This study highlights a new dimension of the challenge posed by stranger-perpetrated TFA. We found that the perpetrators of this form of TFA operate with considerable anonymity, impunity, and unpredictability. These characteristics make such violence difficult to report, limit accountability, and create substantial risk for key populations who are navigating stigma and face heightened vulnerability if their digital health security is compromised.

### Forms and continuities of technology-facilitated abuse

We found that TFA experienced by young adults living with HIV and key populations was cross-cutting and could not be confined to a single category. While participants frequently described experiencing verbal abuse, other forms of TFA primarily revolved the exposure of their sexuality and gender identity, the nature of their work, HIV status, or sharing of explicit photos. This was especially true for gay men and other participants in the LGBTQ+ community, whose identities were outed or threatened with public disclosure of their work or had their nude photos circulated on social media platforms. These cases fall within technology-facilitated sexual violence, in which digital technologies are used to control, shame, monitor, or expose individuals, often targeting their sexual or gender identity [10]. We also documented cases of financial abuse, most of which were linked to the threat or act of exposing participants’ sexual or gender identities.

It emerged from our study that TFA existed along a continuum of informal interpersonal surveillance through social media, in which digital monitoring of social media presence laid the groundwork for coercion, blackmail, and non-consensual disclosure. Acts such as accessing private messages or monitoring social media activity often served as precursors to more harmful behaviours, including cyberbullying and blackmail. This pattern has also been shown in other studies, where surveillance functions as a principal tactic for exerting coercive control and allows perpetrators to maintain a constant presence of their victims [13].

Participants described cases in which digital abuse and online threats escalated into physical violence and threats to life. Previous studies have characterized TFA as a continuation of physical violence and coercive control, in which technologies extend and amplify the same harmful tactics observed in physical and sexual abuse [47]. In the context of this study, where strangers perpetrated TFA was prevalent, participants’ accounts indicate that physical violence is a continuation, rather than a departure, from online abuse.

### Impact of TFA on safety, health, and rights

Participants described experiencing significant and multidimensional harms from TFA, which compromised both their safety and their ability to seek, share, and access health information and care. They described how inadequate legal safeguards, limited digital literacy, and persistent gaps in the criminal justice system left them exposed to ongoing abuse. Although many forms of TFA are formally recognized under the Kenya’s Computer Misuse and Cybercrime Act (Kenya Law, 2022), participants reported receiving little or no meaningful support from police or other institutions [48]. These challenges were particularly pronounced for participants facing heightened stigma, criminalization, and institutional neglect. These failures within the legal and protection ecosystem mirror findings by Brookfield et al., who argue that existing risk-assessment tools and legal responses remain outdated and poorly equipped to address the rapidly evolving nature of TFA [13]. In both contexts, the failure to adequately recognize, respond to, and prevent TFA against key populations reinforces impunity for perpetrators and leaves victims without effective or accessible avenues for redress.

The findings of this study demonstrate the distinct, gendered, and intersectional nature of TFA among young adults living with HIV and key populations in Kenya. While TFA in this context was most frequently perpetrated by strangers, it is predominantly sexuality-based and rooted in broader structures of stigma, criminalization, and social exclusion. These findings underscore the need for Kenyan policy and legal frameworks to evolve in ways that reflect the complex intersectionalities shaping experiences of TFA. In particular, there is a need for explicit recognition of the layered vulnerabilities that render young adults living with HIV and key populations uniquely susceptible to digital harm. Within Kenya’s legal landscape, greater attention must be paid to the multidimensional nature of TFA, its continuity with offline and physical violence, and its role in digitizing pre-existing patterns of gendered and sexuality-based violence.

Our findings affirm that TFA is not merely a technical or cyber issue, but a critical health and human rights concern. For young adults living with HIV and key populations, experiences of TFA cannot be disentangled from the broader social, legal and political environments that criminalize, stigmatize, and marginalize their identities and livelihoods. Addressing TFA effectively, therefore, requires not only strengthened legal protections, but also holistic, rights-based approaches that center the lived realities, agency, and safety of key populations.

### Pathways for action

In light of these findings, coordinated action is required across legal, policy, and community domains, as outlined below in relation to law reform, digital literacy, and community-based justice pathways. Law reform and implementation efforts should prioritize explicit inclusion of TFA in Kenya’s Data Protection Act and the Computer Misuse and Cybercrime Act, alongside survivor-centred reporting and redress mechanisms, and stronger accountability for perpetrators, including those operating anonymously. National digital and health strategies should explicitly acknowledge that TFA disproportionately affects women, gender‑diverse persons, gay men, and other men who have sex with men, and should incorporate targeted, rights‑based protections for these populations.

Concurrently, digital literacy ought to be conceptualized as a core component of the right to education, necessitating its systematic integration into formal and non‑formal education systems. This should include accessible “know your rights” training programs, developed in partnership with community organizations, that equip young people and other at‑risk groups to recognize, prevent, and respond to TFA and related digital harms.

Finally, we call for increased and sustained investment from funders to expand and formalize community paralegal networks and other community‑based justice actors. While the state bears primary responsibility for protecting citizens from TFA, criminalized and stigmatized populations have historically been excluded from, or placed at further risk by, formal justice processes. As civil society organizations have long filled gaps left by the state, our participants identified community paralegal networks as a trusted and critical avenue for recourse, particularly among sexual minorities who fear criminalization, stigma, and secondary victimization within the formal criminal justice system. Consolidating and resourcing these pathways is critical to reducing the current protection gap. However, the most meaningful step toward justice for these populations is decriminalization of same-sex relations, sex work, and transgender identities, which would enable key populations to access formal justice systems safely, without fear of prosecution or persecution. Community paralegal networks are an important measure, but decriminalization remains the foundational goal.

Taken together, these actions - law reform, digital literacy, and investment in community paralegals and decriminalization - provide a concrete roadmap for policymakers, funders, and community actors to move beyond diagnosis of the problem toward solutions that safeguard the dignity, health, and the capacity to act for those most affected by TFA.

### Strengths and limitations

A major strength of this study is its PAR design, which enabled meaningful engagement with populations that are often criminalized, marginalized, and difficult to reach through conventional research approaches. By centering participants’ voices and lived experiences, the study generated contextually grounded insights into technology-facilitated abuse [TFA] that may otherwise remain undocumented.

Conducting the study across four diverse regions of Kenya provided a broad perspective on the dynamics of TFA in contexts with varying levels of economic development and digital access. The inclusion of young adults living with HIV, female sex workers, and gay and other men who have sex with men offered valuable insights into how intersecting related to gender, sexuality, and HIV status shape exposure to and experiences of digital harms.

However, the findings should be interpreted in light of certain limitations. The qualitative design and purposive sampling strategy limit the generalizability of findings beyond the study settings. The study’s focus on specific key populations may not capture the full range of TFA experiences among other groups, such as people who inject drugs. In addition, majority of the participants were affiliated with community-based or civil society organizations, which may not reflect the experiences of those without access to such networks.

Data were based on self-reported experiences, which may be subject to recall bias or social desirability bias. Finally, the study focused on participants with some level of digital access and engagement and may not fully capture the experiences of individuals with limited or no access to digital technologies. Despite these limitations, this study provides robust and timely evidence with important implications for digital health programming, legal reform, and rights-based policy responses.

## Conclusion

This study examined the forms, impacts, and facilitators of TFA among young adults living with HIV and members of key populations in Kenya. It revealed that their experiences of TFA are shaped by intersecting vulnerabilities related to HIV status, sexuality, and gender identity, vulnerabilities that we propose be explicitly recognized in an expanded, intersectional definition of TFA that extends beyond the existing gendered framing. TFA emerged not only as a set of individual online harms, but as a digital extension of offline inequalities, making it a critical public health and human rights concern with serious implications for safety, mental health, and access to care and justice. By framing TFA in this way, this study contributes to emerging scholarship on digital health, rights, and governance in Africa, and underscores that creating safe digital environments is both a technological and a social justice imperative, one that ultimately requires the decriminalization of the identities and practices of those most at risk.

## Supporting information

S1 Checklist Inclusivity in Global Research Questionnaire. (PDF)

S2 Checklist COREQ (COnsolidated criteria for REporting Qualitative research) Checklist. (PDF)
