# Supplementary material for: Navigating technology-facilitated abuse: A participatory action research study with young adults living with HIV and key populations in Kenya
Source: PLOS Glob Public Health. 2026 Aug 14;6(8):e0007060. doi: 10.1371/journal.pgph.0007060 (PMC13475813; doi:10.1371/journal.pgph.0007060)
Supplement: Supplementary file 2 [file pgph.0007060.s002.pdf]

COREQ (COnsolidated criteria for REporting Qualitative research) Checklist

Study Title: *Navigating Technology-Facilitated Abuse: A Participatory Action Research Study with Young Adults Living with HIV and Key Populations in Kenya*

Study design: Qualitative study using in-depth interviews and focus group discussions
Reporting guideline: Consolidated Criteria for Reporting Qualitative Research (COREQ)

Domain 1: Research Team and Reflexivity

Item	Guide Question	Response	Page No.
1	Which author/s conducted the interview or focus group?	Trained researchers, and national research assistants conducted FGDs, IDIs, and KIIs.	6
2	What were the researchers' credentials?	Graduate-level training in social sciences, public health, law and/or human rights.	8
3	What was their occupation at the time of the study?	Academic researchers, NGO staff, and community-based research assistants.	8
4	Was the researcher male or female?	The research team included both male and female researchers.	8
5	What experience or training did the researchers have?	Trained in qualitative methods, participatory action research, trauma-informed approaches, and working with vulnerable populations.	8
6	Was a relationship established prior to study commencement?	CAT members and KPs organizations had pre-existing relationships.	7
7	What did participants know about the researcher?	Researchers introduced themselves as trained qualitative facilitators and allies, emphasizing confidentiality	8
8	What characteristics were reported about the interviewer/facilitator?	Positionality, community membership, and potential biases were discussed in consortium meetings and analytic memos.	8

Domain 2: Study Design

Item	Guide Question	Response	Page No.
9	What methodological orientation was stated?	Transnational Participatory Action Research (T-PAR), qualitative approach.	5
10	How were participants selected?	Purposive and snowball sampling via KPs organizations and CAT networks..	7
11	How were participants approached?	Through trusted KPs organizations, CAT recommendations, and peer networks.	7
12	How many participants were in the study?	109 young adults aged 18–30 across four counties.	7
13	How many people refused to participate or dropped out?	No refusals were reported given the use of trusted networks and organizations	7
14	Where was the data collected?	Data were collected in private, convenient locations agreed upon with supporting organizations.	8
15	Was anyone else present besides the participants and researchers?	No non-participants were present during interviews or focus group discussions.	8

16	What are the important characteristics of the sample?	Participants varied by age, gender, and socio-demographic background relevant to the study focus.	7
17	Were interview guides provided by the authors?	A semi-structured interview guide was developed and used to guide data collection.	8
18	Were interviews/focus groups pilot tested?	The interview guide was pilot tested and refined prior to data collection.	8
19	Were repeat interviews carried out?	No repeat interviews were conducted.	8
20	Were audio or visual recordings made?	Interviews and focus group discussions were audio-recorded with participants' consent.	8
21	Were field notes made during and/or after the interview?	Field notes were taken during and immediately after each data collection session.	8
22	What was the duration of the interviews or focus groups?	Interviews and focus group discussions lasted approximately 60–90 minutes.	8
23	Was data saturation discussed?	Preliminary codebooks were iteratively tested until no new themes emerged.	9
24	Were transcripts returned to participants for comment or correction?	Transcripts were not returned to participants for comment or correction. Validation conducted through CAT and participant workshops.	9

Domain 3: Analysis and Findings

Item	Guide Question	Response	Page No.
25	How many data coders coded the data?	Two coders per transcript; discrepancies resolved by a third researcher.	9
26	Did authors provide a description of the coding tree?	Codebook developed iteratively from analytic memos and consortium consensus.	9
27	Were themes identified in advance or derived from the data?	Themes were inductively derived from the data.	9
28	What software, if applicable, was used to manage the data?	Qualitative data were managed and analysed using Deedose software.	8
29	Did participants provide feedback on the findings?	Validation of the findings conducted through CAT and participant workshops.	9
30	Were participant quotations presented to illustrate the themes?	Anonymized quotes with participant codes, age, gender, and site.	8, 9
31	Was there consistency between the data presented and the findings?	Yes. Verbatim participant quotations and validation workshops with CATs and participants	17, 18
32	Were major and minor themes clearly identified?	Yes, organised by forms of TFA, impacts, coping strategies, and facilitators	10, 13, 16, 17

Statement for manuscript:

"This study is reported in accordance with the Consolidated Criteria for Reporting Qualitative Research (COREQ) guidelines. Page numbers corresponding to each item are indicated in the checklist."
